# Sensor-Based and AI-Driven Ergonomic Seated Posture Detection for Workplace Risk Prevention

**DOI:** 10.3390/s26165120

**Published:** 2026-08-13

**Authors:** Tatiana Teixeira, Guilherme Barbosa, Bruno Areias, Ana Guerra, Maria Covas, Sara Faria, Rita Machado, João Amorim, Luís Ferreira, Beatriz Costa, Júlio Martins, Emanuel Dias, Sérgio Fonseca, Renato Costa, Nilza Ramião

**Affiliations:** 1 INEGI—Institute of Science and Innovation in Mechanical and Industrial Engineering, 4200-465 Porto, Portugal; gbarbosa@inegi.up.pt (G.B.); bareias@inegi.up.pt (B.A.); aguerra@inegi.up.pt (A.G.); mariadaluzcovas@hotmail.com (M.C.); nramiao@inegi.up.pt (N.R.); 2 CeNTI—Centre for Nanotechnology and Advanced Materials, 4760-034 Vila Nova de Famalicão, Portugal; sfaria@centi.pt (S.F.); armachado@centi.pt (R.M.); jamorim@centi.pt (J.A.); 3 Sensing Future Technologies, 3045-508 Coimbra, Portugal; luisferreira@sensingfuture.pt (L.F.); beatrizcosta@sensingfuture.pt (B.C.); 4 Everythink, Lda, 4200-135 Porto, Portugal; juliomartins@everythink.com (J.M.); emanueldias@everythink.com (E.D.); 5 PLUX—Wireless Biosignals, S.A., 1050-059 Lisboa, Portugal; sfonseca@pluxbiosignals.com (S.F.); rcosta@pluxbiosignals.com (R.C.)

**Keywords:** human posture recognition, postural risk, smart office systems, musculoskeletal disorders, office work environment

## Abstract

Background: Work-related musculoskeletal disorders (WMSDs) remain one of the most prevalent occupational health problems worldwide. To prevent the development of these WMSDs in an office space, a chair designed for office monitoring capable of accurately identifying ten representative seated postures and measuring environmental factors was developed and validated. Methods: To evaluate office working conditions, the chair has three embedded Printed Circuit Boards (PCBs): one directed towards seat pressure management, one directed towards environmental measurements and one PCB to manage the entire system. Machine learning approaches were then applied to establish a model that effectively predicts the seated position. The environmental data were also analyzed. Results: The seated position classification presented an accuracy of 80.99% in controlled conditions, while in a real-world context the accuracy was 65.98%. The environmental management showed low errors, except for the PM_2.5_ and PM_10_, with relative errors above 30%. Conclusions: This work presents an initial promising first step for an ergonomic office management solution.

## 1. Introduction

Work-related musculoskeletal disorders (WMSDs) remain one of the most prevalent occupational health problems worldwide and constitute a major cause of work-related disability. According to the World Health Organization (WHO), approximately 1.71 billion people globally are affected by musculoskeletal conditions, which represent the leading cause of reduced work capacity and participation in daily activities [1]. Musculoskeletal pain is also the most common form of non-cancer-related pain and has a substantial impact on productivity, absenteeism, and workers’ quality of life [2].

Office and administrative workers are particularly exposed to WMSDs due to the predominantly sedentary nature of their work and sustained exposure to static postures. In Europe, around 18% of workers report spending more than 7.5 h per day seated, highlighting the extent of prolonged sedentary behaviour in modern work environments [3]. Prolonged sitting, especially when associated with inadequate postures, has been identified as a critical risk factor for the development of musculoskeletal symptoms [4].

The development of WMSDs is multifactorial, resulting from the interaction between individual characteristics, biomechanical demands, organizational factors, and psychosocial conditions. Extended periods of sitting may lead to biomechanical alterations such as hyperkyphosis, hyperlordosis, altered spinal alignment, and shoulder rotation [5,6]. Additionally, reduced movement contributes to muscle stiffness, decreased joint mobility, diminished flexibility, and impaired blood circulation, increasing the risk of lower back pain, neck pain, and other musculoskeletal disorders [4,7]. These effects are often aggravated by ergonomic inadequacies at the workstation, including unsuitable chairs, improperly positioned monitors, non-ergonomic input devices, inadequate lighting, and unfavourable thermal conditions [8].

Ergonomic risk assessment is traditionally performed using methods based on observation, questionnaires, and image analysis. Although widely used, these approaches are limited by subjectivity and dependence on the evaluator’s expertise. Furthermore, they often lack accuracy in measuring postural angles and dynamic movements, tend to focus on isolated or peak postures, and do not adequately capture postural variability throughout the working day [9,10].

In response to these limitations, sensor-based ergonomic assessment methods combined with artificial intelligence (AI) and machine learning (ML) techniques have emerged as promising alternatives. These approaches enable continuous, objective, and quantitative monitoring of workers’ postures, improving the identification of ergonomic risks and supporting preventive interventions [11]. Despite their potential, the implementation of complex monitoring systems in real work environments presents challenges related to data complexity, user comfort and acceptance, external interferences, and the balance between accuracy, cost, and practical applicability [12].

In office environments, pressure sensors integrated into chair seats and backrests offer a non-intrusive and effective solution for postural monitoring. When combined with AI-based classification algorithms, these systems enable rapid and accurate identification of seated postures and associated ergonomic risks, contributing to the prevention of inadequate postures and the promotion of comfort and well-being among office workers [13].

Therefore, the main objective of this study is to develop and validate an artificial intelligence-based system capable of accurately identifying ten seated postures using pressure sensors embedded in the seat and backrest of an office chair, in order to support ergonomic risk assessment and contribute to the prevention of work-related musculoskeletal disorders, while promoting improved comfort, well-being, and postural awareness among office workers.

## 2. Materials and Methods

### 2.1. Posture Identification

To identify the postures most frequently adopted in office environments while seated, a narrative literature review was conducted. Based on this analysis, the ten most common postures were selected, followed by interviews with 20 administrative workers to verify the extent to which the postures identified in the literature corresponded to those actually adopted in their daily activities.

### 2.2. System Architecture Development

The developed system comprises an integrated hardware and software architecture designed to support real-time ergonomic assessment through posture and environmental monitoring. The hardware architecture is based on a three-PCB configuration, including two sensing modules and a central control unit. One PCB is dedicated to postural monitoring, acquiring pressure distribution data from a 32 × 32 cm pressure sensor matrix located on the seat and six individual force-sensitive resistors (FSRs) positioned on the backrest. A second PCB integrates environmental sensors to monitor parameters such as carbon dioxide (CO_2_) (SCD41-D-R2, Sensirion AG, Stäfa, Switzerland), particulate matter (PM_2.5_ and PM_10_) (SPS30, Sensirion AG, Stäfa, Switzerland), volatile organic compounds (VOCs) (SGP41-D-R4, Sensirion AG, Stäfa, Switzerland), nitrogen oxides (NOx) (SGP41-D-R4, Sensirion AG, Stäfa, Switzerland), temperature, and relative humidity (SHT35-DIS-B2.5KS, Sensirion AG, Stäfa, Switzerland).

**Figure 1:**
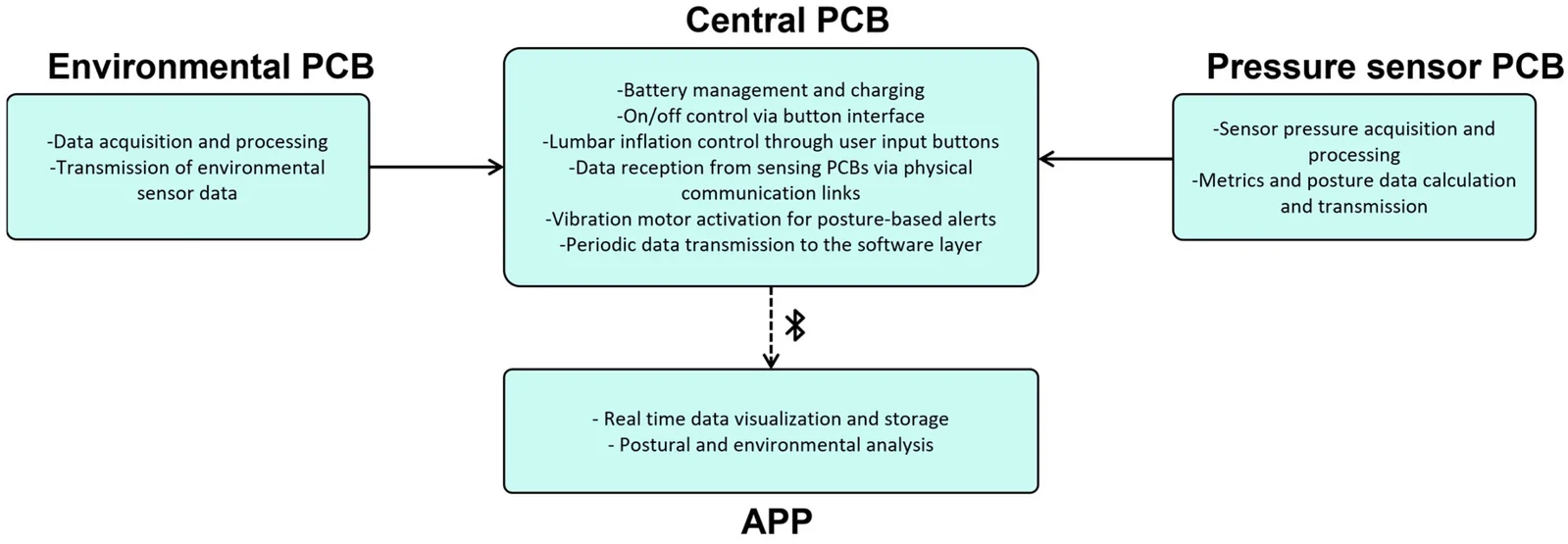
System architecture of the proposed solution, detailing the interaction between hardware modules (postural and environmental sensing units and central control PCB) and the software platform, including data acquisition, processing, and communication (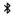 represents Bluetooth connection).

A central PCB acts as a main control and data aggregation unit, receiving inputs from both sensing modules. The postural monitoring component communicates via a low-power serial communication protocol (LPUART), whereas the environmental component communicates via I2C (inter-integrated circuit) (Figure 1).

The software architecture consists of a monitoring application responsible for data reception, storage, and processing. The application provides real-time visualization of posture, ergonomic indicators, and long-term behavioural analysis, including posture distribution and sedentary patterns. A machine learning model based on a decision tree classifier is integrated into the system to classify up to ten different postures based on extracted pressure features.

**Figure 2:**
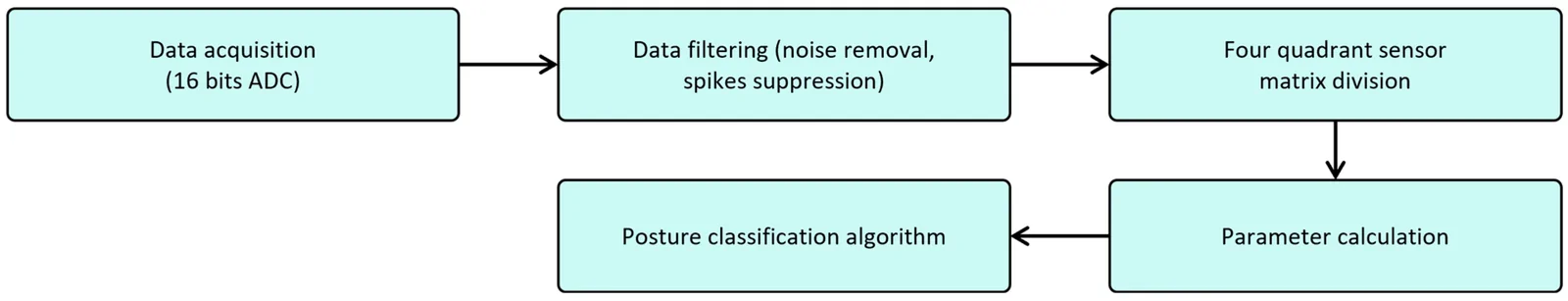
Data processing pipeline for posture classification, including signal acquisition, preprocessing, feature extraction, and classification stages.

In terms of data flow, pressure signals are acquired through a 16-bit analogue-to-digital converter and subjected to a multi-stage preprocessing pipeline, including median filtering and artifact removal. The processed pressure matrix is divided into four quadrants, from which features such as maximum and average pressure, pressure distribution, contact area, and centre of pressure (COP) are extracted. These features, together with backrest sensor data, are used as inputs to the classification model (Figure 2).

**Figure 3:**
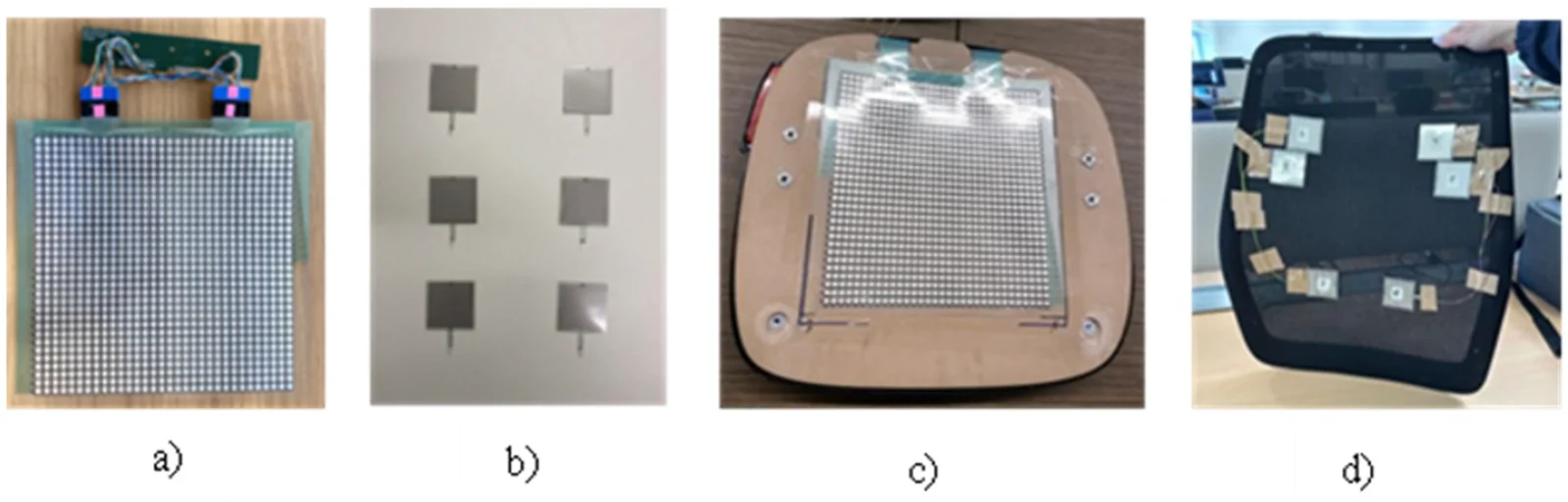
Pressure sensor integration in the prototype chair. (**a**) Pressure sensor matrix and PCB; (**b**) FSR for the backrest; (**c**) pressure sensor matrix at the seat base; (**d**) FSR at the backrest.

The sensing system design prioritizes flexibility, low power consumption, and ease of integration. Force-sensitive resistors were selected due to their robustness, adaptability, and suitability for distributed pressure sensing. The final configuration includes a 32 × 32 cm pressure sensing area in the seat and six additional sensors in the backrest, ensuring comprehensive coverage of user posture (Figure 3).

To ensure signal quality, preprocessing techniques were implemented, including median filtering and noise suppression to remove artefacts caused by cushioning materials and transient pressure variations. Data are stored in a structured SQL database, enabling user-specific tracking and long-term analysis while ensuring data privacy through secure authentication mechanisms.

Various features were extracted from the embedded sensors. These features are listed in Table 1. The feature name is defined on the left, while a short explanation is detailed on the right.

**Figure 4:**
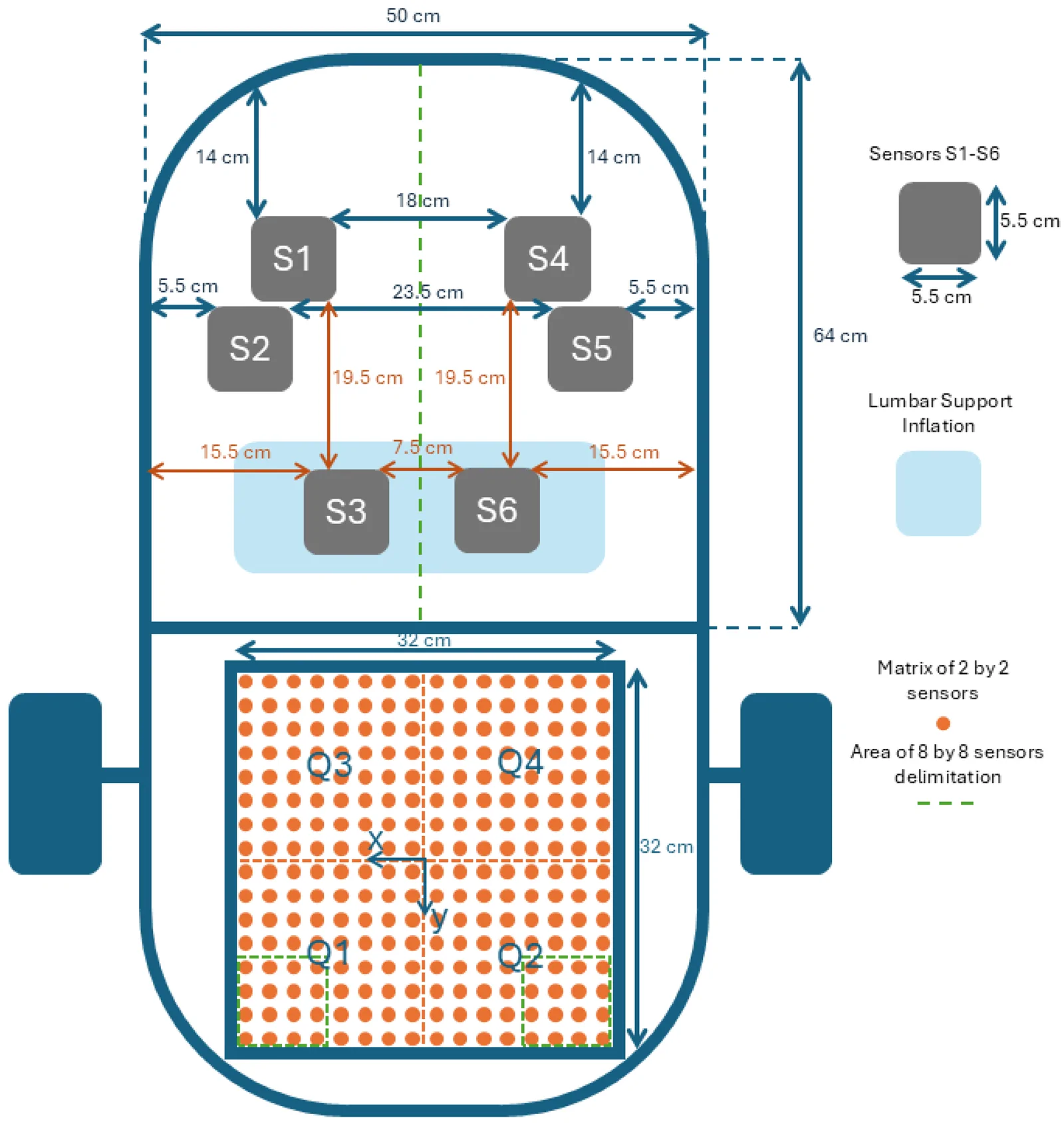
Feature extraction and sensor placement within the chair prototype.

To bring context and further insight into how these features are extracted, a diagram of the chair’s embedded sensors is presented in Figure 4. This diagram presents the sensors’ placement and configuration. The four different quadrants defined in the pressure matrix at the seat base are signalled as Q1, Q2, Q3, and Q4. The back sensor positions are defined as S1 through S6. The inflation mechanism is represented in a lighter blue. The seat pressure matrix is represented through sets of 2 by 2 sensor position matrices with orange circles. Meanwhile, the area of 8 by 8 cm present in features Q1_A8×8 and Q2_A8×8 is delimited by a green line. The x and y axes are defined at the centre of the matrix, with the *x*-axis pointing from Q4 to Q3, while the *y*-axis points from Q4 to Q2.

### 2.3. Preliminary Experiments

With the aim of establishing a methodology for postural risk assessment and defining maximum sedentary exposure times to support system alerts, a targeted analysis was conducted using previously collected data. Five participants were randomly selected, and pressure values (Pa) were extracted from frame-by-frame pressure mat data for each posture. To ensure comparability, an equal number of frames per posture was considered, followed by the calculation of individual and overall mean pressure values.

Additionally, the centre of pressure was computed based on the mat geometry (465 × 465 mm for the backrest sensors; 320 × 320 mm for the seat sensors), enabling spatial characterization of pressure distribution. The ten selected postures were then characterized using both quantitative pressure data and observational analysis supported by lateral-view photographic records, assessed according to the ErgoCheck methodology ISO/TR 12295:2014; ISO 11226:2000 [14,15].

This integrated analysis allowed the definition of postural risk levels and associated recommendations, including time thresholds for sedentary exposure. These thresholds were further supported by a state-of-the-art review, leading to the implementation of two alert types: (i) incorrect posture alerts and (ii) prolonged sitting alerts. Pressure maps were also analyzed to characterize user positioning and contact distribution.

### 2.4. Algorithm Development

A decision tree classifier was trained to map sensor data into posture classes. Hyperparameters controlling model capacity (maximum depth, minimum samples per split/leaf, split criterion, maximum features, maximum leaf nodes, minimum impurity decrease, alpha parameter for minimal cost-complexity pruning, quality criteria for optimization, including Gini and Shannon entropy) were optimized via grid search within cross-validation (CV).

CV used the GroupKFold function in sklearn to ensure that participants were not repeated in any of the training groups (training, validation, and testing). Complexity control was enforced through cost-complexity pruning with comparable validation performance, reducing overfitting and improving interpretability. CV was performed both with inner validation and outer validation to separate training from validation data, and additionally to separate the testing data as well. Three metrics were used to evaluate the model: accuracy, balanced accuracy, and the F1-score.

A grid search procedure was applied to identify the most suitable combination of hyperparameters for the decision tree classifier. The evaluated parameters included the splitting criterion (Gini and Shannon entropy), the minimum number of samples required to split an internal node (2 and 5), the minimum number of samples per leaf (1 and 5), and different class weighting strategies to address class imbalance. The maximum depth was fixed at 8, the maximum number of leaf nodes was limited to 120, and the number of features considered at each split was set to sqrt. Additionally, the grid search tested different values of the cost-complexity pruning parameter (ccp_alpha), while keeping the minimum impurity decrease fixed at 0.

The final model was configured using the best hyperparameters identified during the optimization process, including a maximum tree depth of 8 levels, a maximum of 120 leaf nodes, and a minimum of 5 samples per leaf. These settings were selected to control the complexity of the tree, reduce the risk of overfitting, and improve the model’s ability to generalize to unseen data. Internal nodes represented decision rules based on the input features, whereas terminal nodes corresponded to the predicted classes. This implementation represented the learned decision rules as conditional statements, enabling direct interpretation and practical deployment of the trained model.

### 2.5. Real-World Validation and Analysis

#### 2.5.1. Participants

For the validation of the first prototype of the sensorized chair, an experimental study was conducted with office workers under prolonged usage conditions. The sample consisted of 10 employees from a research institute, randomly selected from individuals with predominantly sedentary activities in an office environment, who used the prototype for at least 6 h per day over 5 consecutive days under representative real-use conditions. The sample comprised 5 male and 5 female participants, with a mean age of 35.20 ± 10.53 years, mean body weight of 71.95 ± 10.02 kg, and mean height of 168.50 ± 7.53 cm. The average body mass index (BMI) of the sample was 25.31 ± 2.91 kg/m^2^. Data collection was carried out between 9 March and 15 May 2026. Participation was voluntary, and all individuals provided informed consent prior to the start of the study. The principles of confidentiality and data protection were ensured in accordance with European regulations, with only the information strictly necessary for sample characterization and result analysis being collected, namely sex, age, height, and weight.

Additionally, controlled tests were conducted to validate the posture detection system, in which participants performed the predefined postures in a structured manner. This procedure enabled the evaluation of the algorithm’s posture classification accuracy and its rate of correct identification.

#### 2.5.2. Data Collection

Posture detection reliability was assessed through a daily 30 min verification protocol. Under researcher supervision, participants performed 10 predefined postures, each maintained for 1 min to ensure stabilization and accurate system acquisition. For each posture, agreement between the software classification and the actual posture was recorded, allowing calculation of identification accuracy per posture and per participant.

The protocol was conducted under two conditions: without and with lumbar support inflation. The actuation system consisted of a pneumatically controlled lumbar bladder integrated into the backrest a Piezoelectric Disc Pump UXPB5400200A (The Lee Company, Westbrook, ME, USA). Vibration Motors JYC1234 (JIE YI Electronics Limited, Shenzhen, China) were incorporated to provide vibrotactile feedback indicating prolonged sedentary behavior and incorrect postures. Pressure was individually adjusted to a comfortable level prior to testing. Identical procedures were maintained across conditions to enable direct comparison.

Environmental parameters (temperature, relative humidity, volatile organic compounds, respirable and inhalable particles, nitrogen oxides, and carbon dioxide) were measured using two systems: an internal PCB integrated into the prototype and an external monitoring unit placed on the desk for comparison. The sensors used were SCD41/SCD4x (CO_2_), SGP41 (VOC/NOx), SHT35/SHT3x-DIS (temperature and humidity), and SPS30 (particulate matter). Measurements were collected at two fixed times per day under consistent conditions, enabling assessment of inter-system agreement and the influence of sensor placement. Environmental parameters were not used as input features for posture classification; they were collected to characterize the workplace environment and assess the integrated monitoring functionality.

A technology acceptance questionnaire was developed, comprising two sections. Section A included sociodemographic questions to characterize the sample and contextualize the results. Section B consisted of 24 Likert-scale items assessing comfort, system usability, and interface (*Ergonomia App*) usability. Additionally, Section B included four open-ended questions to collect qualitative feedback and improvement suggestions, complementing the qualitative analysis with user perceptions and recommendations.

#### 2.5.3. Data Analysis

The data collected during the experimental period were subjected to statistical treatment using descriptive statistics methods, namely through the calculation of measures of central tendency and dispersion, including means and standard deviations, to characterize the overall behaviour and variability of the variables under analysis.

The evaluation of the postures adopted by the participants was carried out based on the rate of correct posture identification. These results were subsequently compared with the accuracy of the developed algorithm, enabling a quantitative assessment of the performance of the posture recognition system. This approach allowed the identification of potential systematic deviations and discrepancies between the automatic classification performed by the software and the established reference, thereby contributing to the validation of the algorithm under real-world operating conditions.

Regarding environmental parameters, the analysis of weekly average values was conducted, determined individually for each participant, based on data acquired by the developed software and the environmental monitoring unit, as previously described. These values were compared with normative reference values applicable to office work environments, to assess the compliance of the monitored conditions. The reference limits considered are summarized in Table 2.

To accurately measure the efficiency of the sensors applied for environmental measurements, both the measurements acquired within the chair and outside the chair are subject to a relative error calculation (*Er*). This error is calculated from the difference in sensor readings from environmental variable values between the internal PCB embedded within the chair (*Vint*) and the external monitoring unit at the top of the desk (*Vext*), divided by the variable values obtained from *Vext*. This is represented in Equation (1).

(1) $$Er\left(\%\right)=\frac{Vint-Vext}{Vext}*100$$

## 3. Results

### 3.1. Posture Identification

**Figure 5:**
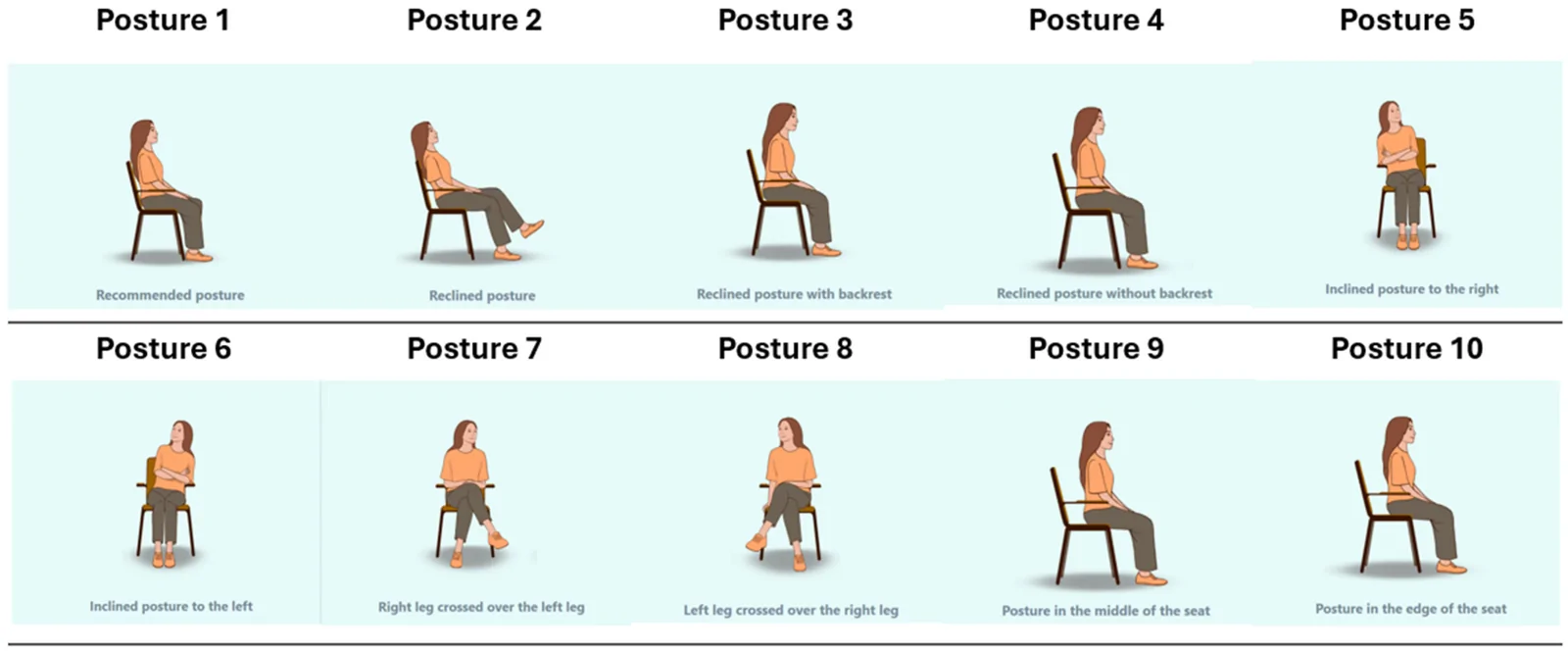
Postures identified and used in the study, including their classification for ergonomic assessment.

**Figure 6:**
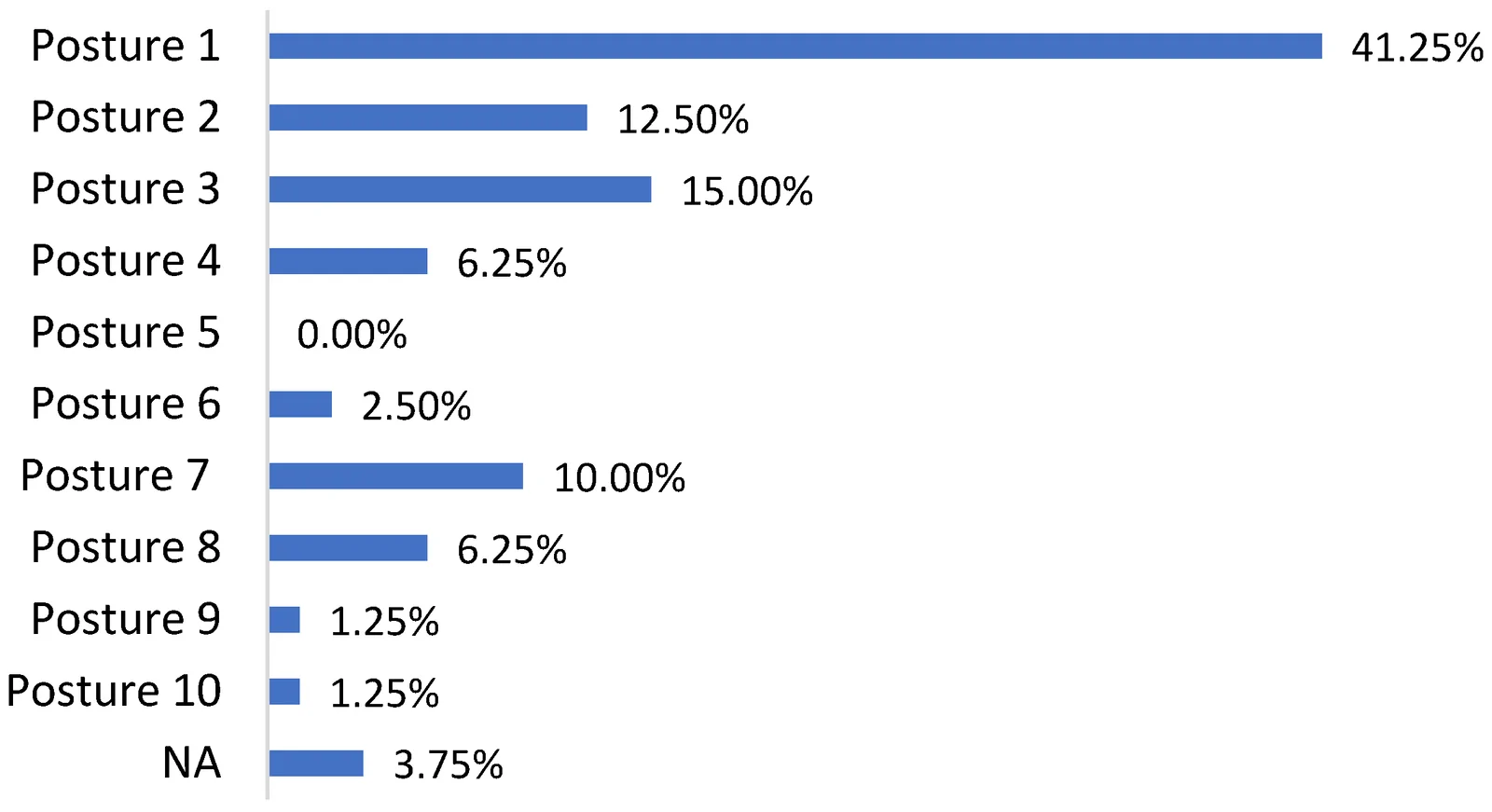
Percentage distribution of postures adopted by participants (*n* = 20), indicating the relative frequency of each posture; (NA represents cases that do not fall into any of the defined posture categories).

Based on the available scientific evidence, ten predominant sitting postures adopted by office workers were identified (Figure 5) [20,21,22,23,24]:

To validate the identified postures, 20 participants were surveyed regarding the postures they most frequently adopted throughout the workday, as shown in Figure 6.

The analysis revealed that Posture 1 was the most frequently adopted, accounting for 41.25% of the observations. This was followed by Posture 3, with a frequency of 15.00%. The third most frequent posture was Posture 2, representing 12.50%, followed by Posture 7, recorded in 10.00% of the cases. The remaining postures showed significantly lower frequencies, with Posture 5 notably not being observed in any of the analyzed instances. Nevertheless, Posture 5 was retained in the classification system because it represents right-side lateral trunk inclination, which is the symmetrical counterpart of Posture 6 (left-side lateral trunk inclination), allowing the model to account for bilateral postural deviations.

### 3.2. System Architecture Development

The developed system successfully integrates multimodal sensing, processing, and feedback functionalities into a cohesive architecture capable of real-time ergonomic assessment. The three-PCB hardware configuration demonstrated reliable acquisition and transmission of both postural and environmental data, ensuring synchronized monitoring of user posture and surrounding conditions.

The pressure sensing subsystem enabled detailed characterization of user posture through high-resolution spatial data obtained from the seat matrix and backrest sensors. The implemented preprocessing pipeline effectively reduced noise and artefacts, resulting in stable and interpretable pressure patterns suitable for feature extraction and classification.

The communication between sensing modules and the central control unit, as well as between hardware and software layers via Bluetooth, proved robust under the defined acquisition frequency (1 Hz), supporting continuous monitoring without significant data loss. Additionally, the system demonstrated effective coordination between data acquisition and actuation, enabling timely feedback through vibration alerts and adaptive control of the inflation mechanism.

The software platform provided comprehensive visualization and analysis capabilities, including real-time posture identification, ergonomic scoring, and long-term tracking of user behaviour. The integration of the decision tree classifier allowed the system to automatically interpret pressure patterns and classify user postures, supporting actionable feedback for posture correction.

Furthermore, the modular architecture facilitated the integration of environmental monitoring alongside postural assessment, reinforcing the system’s holistic approach to ergonomics. The combined functionality of sensing, processing, and actuation highlights the feasibility of the proposed system as a practical tool for workplace ergonomic monitoring and user awareness.

### 3.3. Preliminary Experiments

Table 3 presents the three levels of risk established for each posture. It was observed that only Posture 1 can be classified as acceptable, as it ensures full support of the back and arms, allowing adequate contact between the participant and the chair backrest. Postures 3, 5, and 6 were classified as moderate risk, since, although they involve forward and lateral trunk inclinations, respectively, they still enable the participant to support the arms on the desk or on the chair armrests. Despite representing postures that may lead to increased loading in the lumbar region and alterations in spinal curvature, these effects may be partially mitigated by the availability of arm support.

The remaining postures were classified as high risk. This classification is justified by the absence of back support (Postures 4, 9, and 10), the involvement of lumbar spine flexion (Postures 2, 4, 9, and 10), and the potential restriction of blood circulation in the thighs due to increased pressure (Postures 7 and 8).

To identify recommended durations for maintaining a given posture and for prolonged sedentary behaviour, with the aim of minimizing the risk of musculoskeletal discomfort and the development of work-related disorders associated with sitting, maximum allowable durations were established for each posture based on its risk level. This process resulted in the definition of two types of alerts: (i) posture-related alerts, triggered when the user remains for an excessive period in a posture associated with higher ergonomic risk, and (ii) sedentary behaviour alerts, activated when the user remains continuously seated for a period considered excessive, regardless of the posture adopted.

For postures classified as acceptable risk, a maximum continuous duration of 120 min is recommended, followed by a 10 min break involving light activity (e.g., walking). Alternatively, shorter breaks every 20–30 min or a 10 min break after every 30 min of work are advised [14,25].

For postures classified as moderate risk, it is recommended that posture duration should not exceed 15 min, with an emphasis on alternating with acceptable postures. Frequent breaks should also be implemented, consistent with the recommendations for acceptable postures [25,26,27].

Finally, for high-risk postures, the maximum permissible duration should not exceed 4 min, due to the increased likelihood of biomechanical strain and injury [14,26,27].

### 3.4. Algorithm Development

During the algorithm development and training, the dataset was divided into several fold subsets for training and validation. Table 4 presents the number of participants for each set as an example for one fold.

The algorithm development included a class that represented an empty chair; however, this class was not considered during the evaluation process. For evaluation purposes, cross-validation was applied through every fold; each of the entries was classified both for training and validation. The results for validation metrics in every fold were analyzed jointly. The number of samples per class analyzed is presented in Table 5.

During the fine-tuning of the developed algorithm, a decision tree was tested via grid search with several hyperparameters, as presented in Table 6. A decision tree algorithm represents lower complexity and increased feasibility in microcontroller applications. This simpler approach provides computational advantages over more complex algorithmic implementations that fail to adapt in settings with limited computational power. The aggressive and conservative tuning strategies differ in how they regulate tree growth. The criterion uses either Gini or Shannon entropy in the aggressive strategy, while the conservative strategy uses only Gini. Maximum depth, minimum samples split, and minimum samples leaf control tree complexity, with lower values producing larger, more flexible trees and higher values yielding simpler, more generalizable models. Maximum leaf nodes further limit model complexity, while minimum impurity decrease prevents splits that provide only marginal improvements. Overall, the aggressive strategy favours flexibility and lower bias, whereas the conservative strategy prioritizes interpretability, robustness, and reduced overfitting.

**Figure 7:**
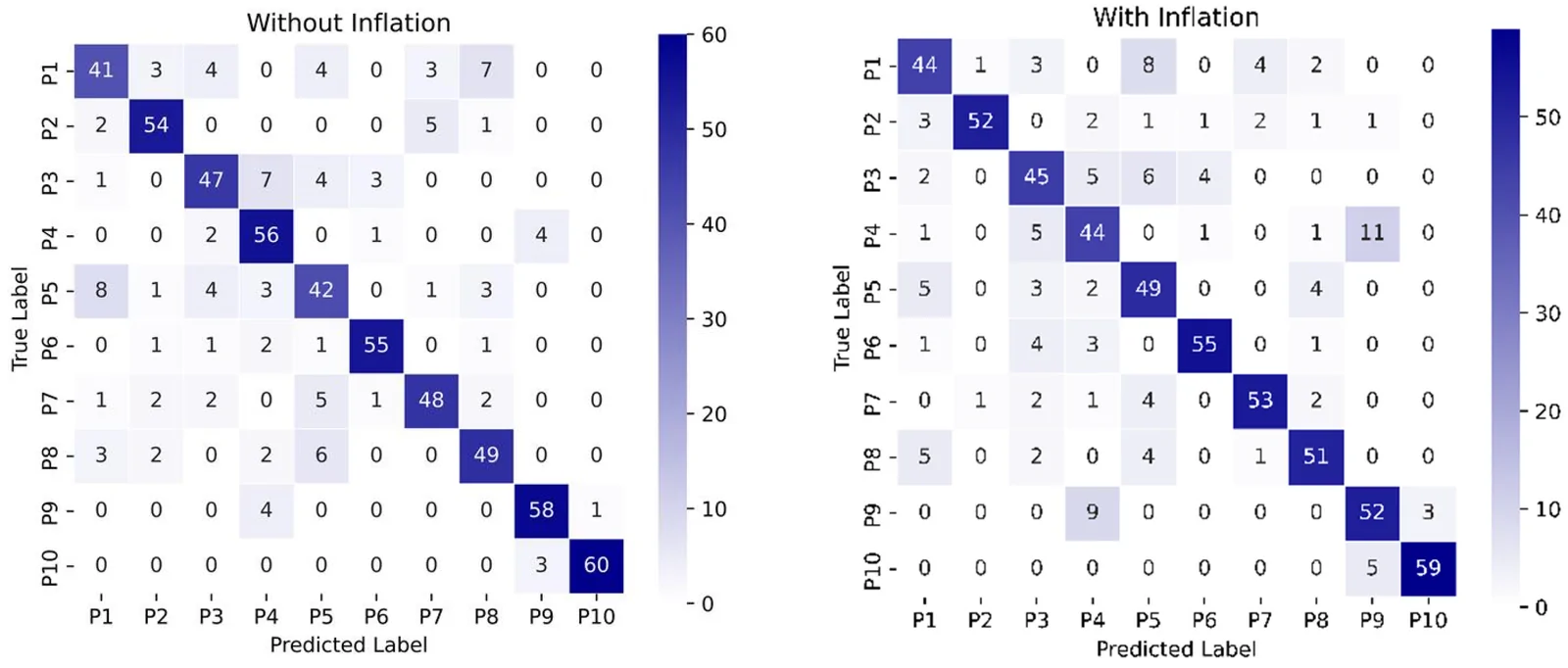
Confusion matrices showing the differences in classification results when inflation is applied versus when it is not applied.

Figure 7 presents the confusion matrices obtained during the testing phase of the algorithm, evaluated under two distinct conditions: the model without inflation and the model with inflation. In both cases, a consistent pattern of high accuracy is observed along the main diagonal, indicating that the classifier demonstrates a robust ability to correctly identify most postures.

Misclassifications occur between postures with similar pressure-distribution patterns, most notably across positions with inflation and without inflation, between P4-P9, P5-P1, P7-P5, and P10-P9. Postures P4, P9, and P10 are particularly difficult to differentiate because they share reduced or absent backrest support and differ mainly by subtle anterior shifts in the pelvis on the seat. These small forward displacements generate highly overlapping pressure-distribution patterns in the seat matrix, limiting the discriminative capacity of features such as CoP_X, CoP_Y, quadrant pressure ratios, and contact area. Additionally, the difference in pressure signals between these positions is not appropriately translated into the features defined through Q1–Q4. The P5 is similar to the P1, except for a slight shift in the trunk towards the right. The differentiation between pressure readings at sensor positions S1, S2, S3 in the right and S4, S5, S6 in the left does not suffice for proper differentiation between these positions. The crossing of the right leg over the left leads to a slight inclination of the trunk to the right, which makes postures P5 and P7 also have coinciding patterns in the backrest pressure signals. The additional features Q1_A8×8, Q2_A8×8 of the front quadrants to differentiate leg crossing, giving precise pressure readings at the left and right edges of the chair, are not enough to accurately differentiate between these two positions. As a result, the current feature set may not fully capture the gradual anterior–posterior displacement between these posture classes, contributing to misclassifications observed in the confusion matrices. Future work should explore more sensitive descriptors of anterior seat displacement, temporal pressure-transition features, and/or additional sensing near the front edge of the seat.

Postures such as the P4 and P3 also exhibit some mismatch; this is understandable given that the only mismatch between these positions is related to the lack of lumbar support in P4. The differentiation in pressure readings from sensors at positions S3 and S4 is insufficient. This mismatch is also noticeable between P1 and P7, and between P1 and P8. The only difference between P7 and P1, jointly with P1 and P8, is due to the slight inclination of the trunk to either the right or left. This demonstrates an inherent difficulty in distinguishing postures that generate comparable load profiles on the seat and backrest. Noticeably, the distinction between the P1 and P8 exhibits better results relative to the P1 and P7. Curiously, although the P8 and P6 positions are also relatively similar. The crossing of the left leg leads to a slight inclination of the trunk to the left. There is no clear mismatch between these positions in the results. The same happens for the positions P1 and P6, although there is only a slight mismatch with the inclination of the trunk towards the left in P6. The detection system appears to have correctly distinguished between the two positions. This indicates that the sensors on the left side of the chair, in positions S4, S5, S6, are a better fit for this detection task.

Table 7 presents the results of the position classification algorithm with inflation and without inflation with the aggressive fine-tuning approach. Overall, the accuracy is 80.99%, the accuracy without inflation is 82.13%, while the accuracy with inflation is 79.87%. Position detection without inflation yields better overall accuracy. The conservative fine-tuning approach obtained 79.90% accuracy; this exemplifies how the aggressive fine-tuning with further generalization, flexibility, and resulting lower bias leads to better results.

Specifically, inflation improves the chair’s performance across all metrics at positions P5, P7, and P8, while decreasing performance on all metrics for P3, P4, P6, P9, and P10. It appears that postures without a backrest, P4, P9, and P10, have better performance without inflation. This is due to a bias introduced by inflation, where the back sensors assume there is contact when there is not. If we compare the confusion matrix between inflation and without inflation, P4 is mistakenly classified as P3 five times with inflation and only two times without inflation. This indicates that the model assumed there was lumbar support when this was not the case. This could be due to a miscalibration of sensors in the back of the chair, in positions S3 and S4, given higher inflation, which deforms the fabric where the sensors are embedded. Curiously, postures P7 and P8, where the main point for posture validation is mostly based on the pressure mat at the bottom of the chair, have better performance without inflation. Although the positions P7 and P8 are differentiable through the seat pressure mat readings, the better performance without inflation could be due to the slight inclination of the trunk between these positions, which relies on the readings from back sensors in positions S3 and S4, which are negatively influenced by inflation.

**Figure 8:**
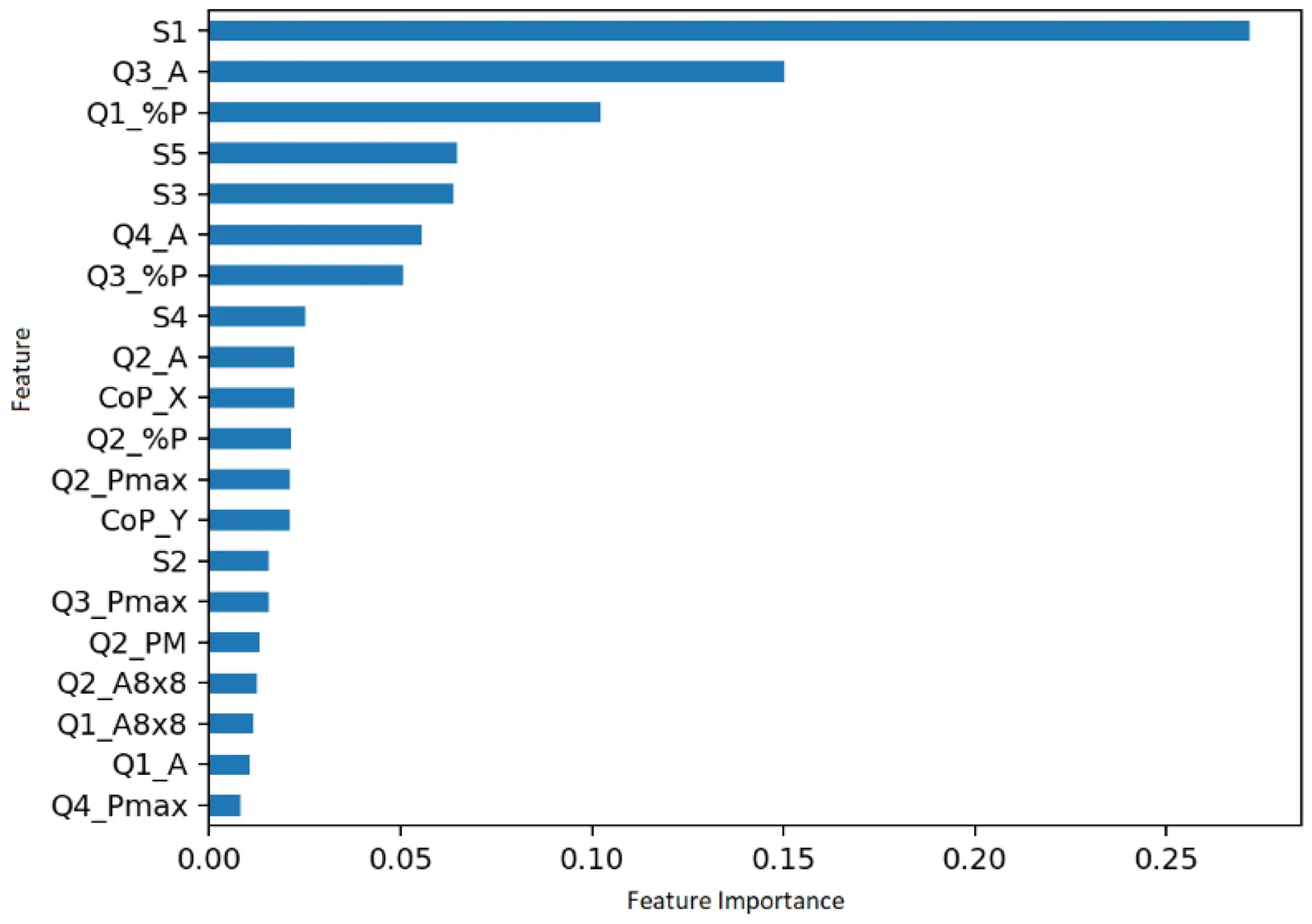
Importance scores of the top 20 features, illustrating their relative contribution to the classification model.

Figure 8 presents the analysis of the 20 most important features, highlighting the variables that contributed most significantly to the discriminative performance of the model. Sensor in position S1 was the most important, and it is in the thoracic region on the backrest of the chair. Among these, several feature groups stand out due to their biomechanical relevance and their ability to capture subtle variations in seated posture. The S1–S6 sensors, distributed across the backrest, capture variations in trunk alignment and contact pressure along the upper, middle, and lower back. Their contribution is particularly relevant for distinguishing postures involving forward leaning, reclined sitting, or lateral asymmetries, as changes in backrest contact patterns are directly reflected in these sensors.

In addition to the backrest sensors, the model relies heavily on features derived from the seat quadrants. The Q1–Q4%P variables represent the percentage of total pressure distributed across each seat quadrant, providing a global overview of how body weight shifts during different postures. Complementing this, the Q1–Q4A features quantify the contact area in each quadrant, reflecting how the user’s body interacts with the seat surface and enabling the detection of postures that modify the extent of contact, such as sitting on the seat edge or shifting laterally.

The Q1–Q4Pmax variables capture the maximum pressure recorded in each quadrant, which is particularly useful for identifying asymmetric or high-load postures. Meanwhile, the Q1–Q4Pmed features represent the average pressure in each quadrant, contributing to the detection of more subtle posture variations that may not produce localized peaks but still alter the overall pressure distribution.

Finally, the Q1–Q2A8×8 features quantify the contact area in the anterior-lateral region of quadrants 1 and 2, offering additional spatial resolution that helps differentiate postures involving forward displacement or partial off-loading of the seat, such as when the user is sitting with one leg crossed.

### 3.5. Real-World Validation and Analysis

**Figure 9:**
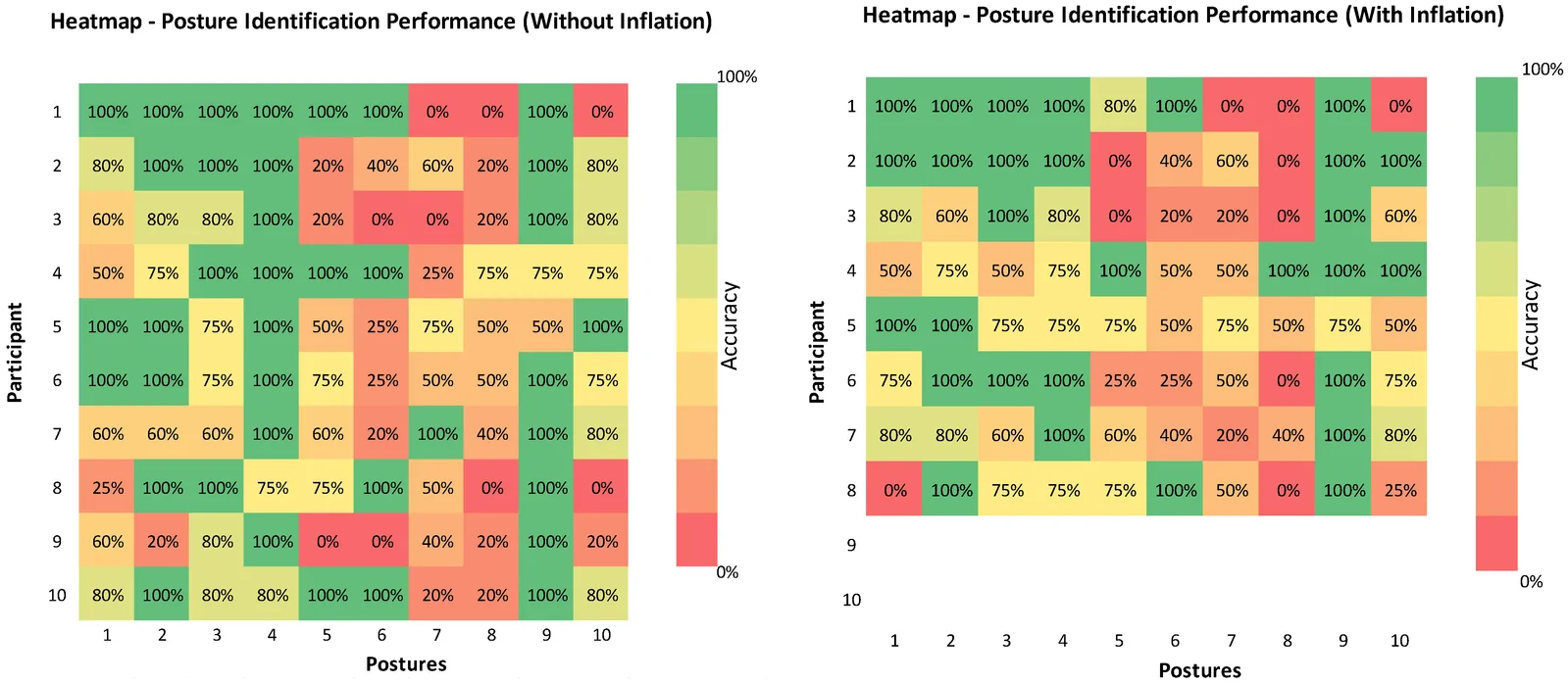
Participant-wise posture identification performance, presented as accuracy heatmaps for models with and without inflation, highlighting variations across postures.

For real-world validation and analysis, an additional subset of 10 participants was used. The data from these 10 participants were not included in the algorithm development. The results revealed variable performance in posture identification, with accuracy rates depending on both posture type and inter-user variability. Overall, the posture classification in real-world settings has an accuracy of 65.98%; with inflation, the accuracy is 65.83%, and without inflation, the accuracy is 66.09%. Postures characterized by more distinct pressure distribution patterns (e.g., Postures 1, 4, and 9) consistently achieved high accuracy values, often reaching 100% across multiple participants. In contrast, postures with similar biomechanical characteristics, particularly those involving comparable trunk inclinations or partial support conditions (e.g., Postures 5, 6, 7, and 8), demonstrated lower and more inconsistent classification performance, with accuracy values frequently dropping below 50% for certain participants (Figure 9). The current classifier was developed as a closed-set model, meaning that it assigns each input sample to one of the ten predefined posture classes. Consequently, unlisted, transitional, or mixed postures are not explicitly recognized by the system and may be classified as the most similar predefined posture based on the extracted pressure features. This limitation is particularly relevant in real-world use, where users may adopt spontaneous postures that do not fully correspond to the predefined set. Future work should include an “unknown/other” class or confidence-based rejection mechanism to identify postures outside the trained classification set.

A detailed examination of the heatmaps further highlights considerable inter-individual variability. Some participants (e.g., Participants 1 and 10) exhibited consistently high recognition rates across most postures, suggesting stable and well-defined pressure patterns. Conversely, other participants (e.g., Participants 8 and 9) presented substantial fluctuations, with several postures showing low or even null identification rates. This variability may be attributed to differences in anthropometric characteristics, seating habits, and interaction with the chair, which directly influence sensor readings.

Regarding the effect of the lumbar inflation system, a comparative analysis between conditions (with and without inflation) indicates a limited overall impact on algorithm performance. While slight improvements can be observed for certain postures and participants, particularly in stabilizing pressure distribution in the backrest, these gains are not consistent across the dataset. In some cases, the inflation condition even introduced additional variability, possibly due to changes in contact areas and pressure redistribution that were not fully captured by the classification model.

Overall, these findings suggest that although the proposed system demonstrates strong potential for detecting clearly distinguishable postures, its performance decreases in scenarios involving subtle postural differences. This highlights the need for further optimization of both the sensing configuration and the classification algorithm to enhance robustness and generalizability across users and posture types. Furthermore, data from participants 9 and 10 were not included due to a malfunction in the inflation system, which prevented the acquisition of their measurements.

**Figure 10:**
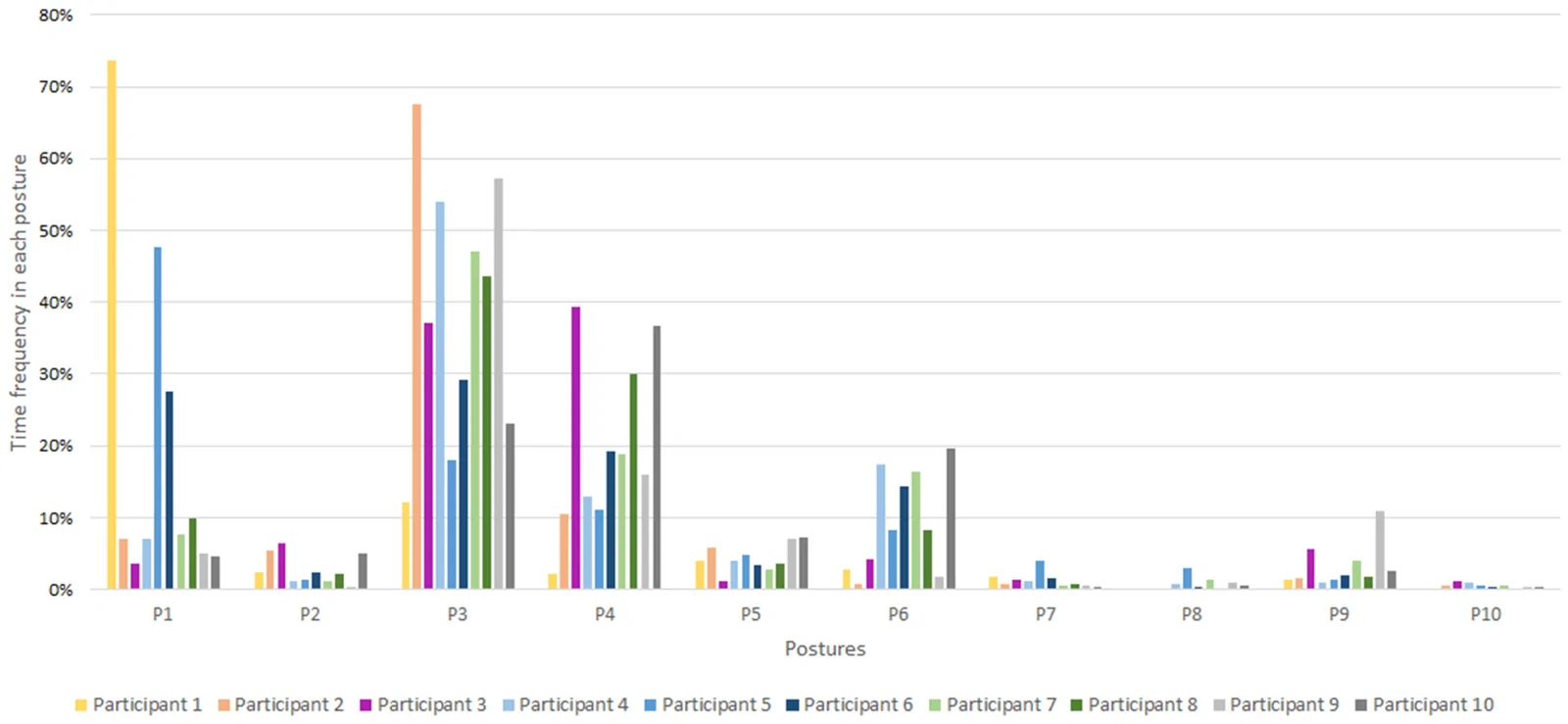
Naturalistic posture distribution during the 5-day workplace use period. Percentage of time spent in each of the ten predefined postures by each participant during the week of prototype use in an office environment.

In addition to the supervised posture-validation protocol, the data collected during the 5-day workplace use period were analyzed to characterize the naturalistic distribution of sitting postures. The percentage of time spent in each posture showed considerable variability between participants, indicating that posture adoption during office work is highly individual and influenced by user-specific behaviour and task demands (Figure 10). Overall, the least frequently adopted postures were Postures 7, 8, 9 and 10, which generally represented only a small proportion of the total sitting time. In contrast, Postures 3 and 4 were frequently observed in several participants, suggesting a tendency towards forward-leaning sitting patterns during computer-based office work. Although Posture 1 was defined as the most recommended posture from an ergonomic perspective, it was not consistently the most prevalent posture across participants. This result is particularly relevant because it indicates that the presence of posture-related alerts was not sufficient, by itself, to ensure sustained adoption of the recommended upright posture. Several factors may explain this finding, including individual comfort preferences, habitual sitting behaviour, the nature of the work tasks performed, possible adaptation to or habituation to alerts, and the fact that users may perceive forward-leaning postures as more functional during desk-based activities. These results suggest that future developments should explore more personalized and adaptive feedback strategies, rather than relying only on fixed alerts for posture correction.

#### 3.5.1. Environmental Parameters

Regarding the environmental monitoring module, a good overall consistency was observed between internal and external measurements, although some differences were identified. These discrepancies can be explained by the influence of the microenvironment within the chair and the specific placement of the sensors. Certain parameters, such as particulate matter, demonstrated higher sensitivity to sensor positioning, leading to greater variability in the recorded values.

**Figure 11:**
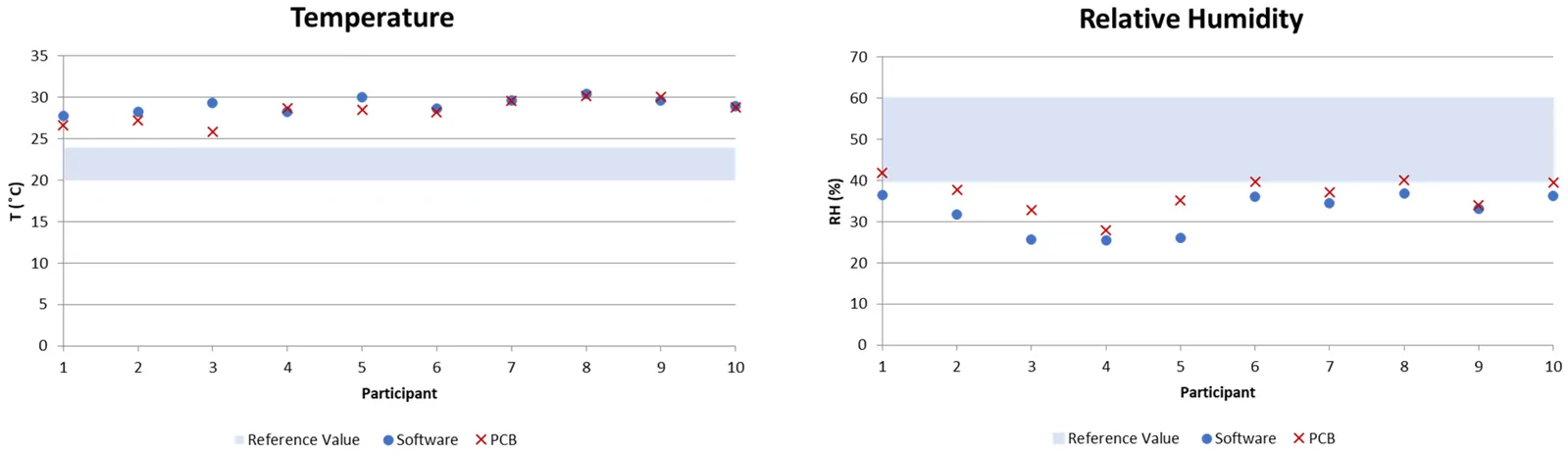

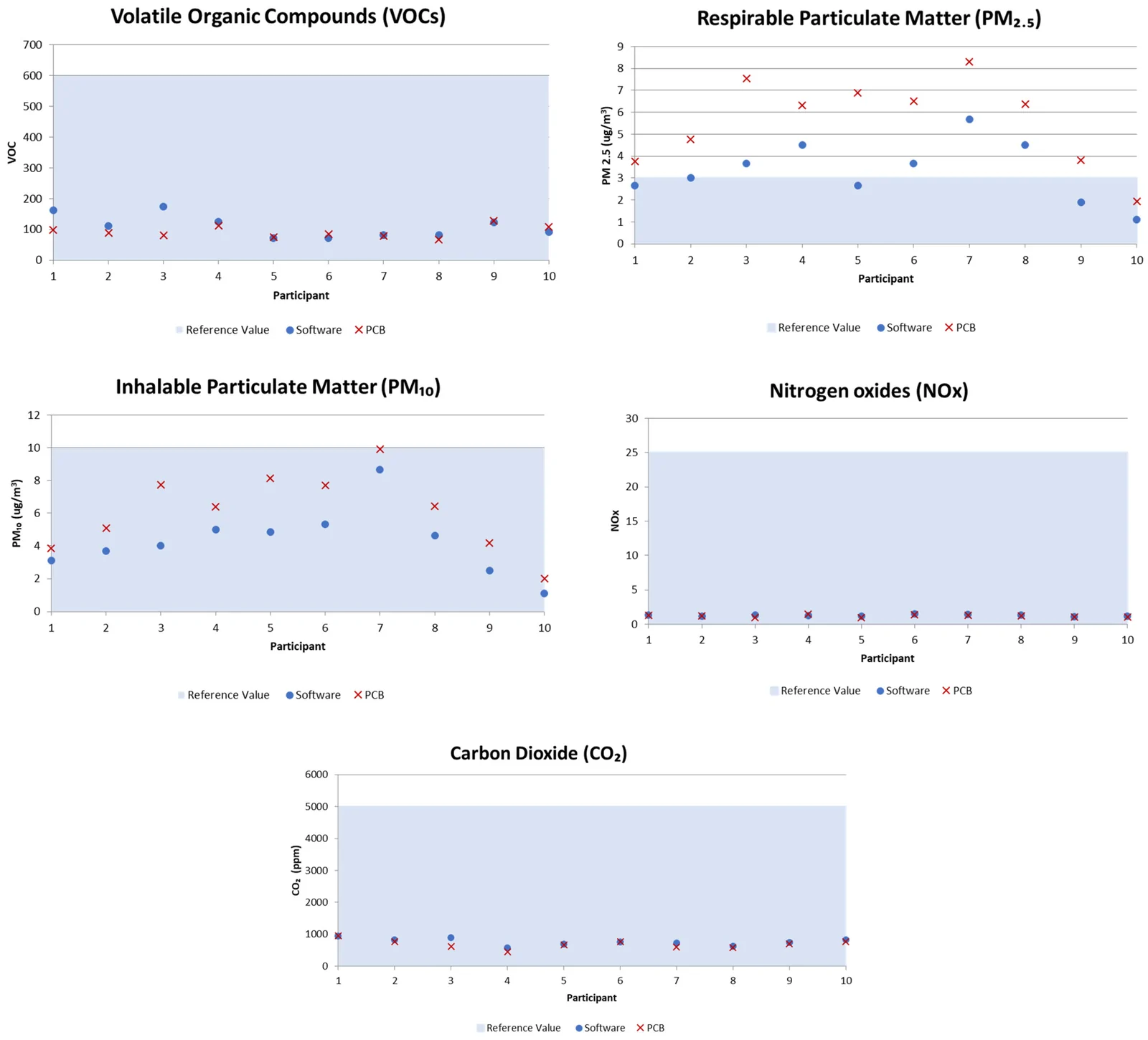
Measured environmental parameters for each participant, including temperature, relative humidity, volatile organic compounds (VOCs), particulate matter (PM_2.5_ and PM_10_), nitrogen oxides (NOx), and carbon dioxide (CO_2_), with comparison to reference values obtained from software and PCB sensors.

A more detailed analysis of the results shows that temperature and CO_2_ measurements remained relatively stable across participants, with values consistently within the reference range, indicating that the system provides reliable readings for these parameters under typical indoor conditions. Relative humidity presented moderate deviations between internal and external measurements, suggesting a localized influence of the seating microenvironment, possibly due to body heat and limited air circulation around the sensor location (Figure 11).

In contrast, particulate matter (PM_2.5_ and PM_10_) exhibited higher variability, particularly in the PCB sensor readings, which were systematically higher than those recorded by the external comparison system. This pattern reinforces the sensitivity of particulate measurements to sensor positioning and airflow conditions, indicating that localized accumulation or resuspension of particles near the chair may affect the readings. Similarly, VOC measurements showed some dispersion, although remaining within acceptable reference limits, suggesting minor influence from environmental or user-related factors.

Nitrogen oxides (NOx) displayed consistently low values with minimal variation between measurement systems, indicating both low environmental concentrations and stable sensor performance for this parameter. The *Er* for each of the variables analyzed is presented in Table 8.

Overall, these findings suggest that while the proposed environmental monitoring module demonstrates robust performance for stable parameters such as temperature, CO_2_, and NOx, additional calibration and optimization may be required for parameters more sensitive to local environmental conditions to improve measurement accuracy and consistency across different usage scenarios.

#### 3.5.2. Technology Acceptance

**Figure 12:**
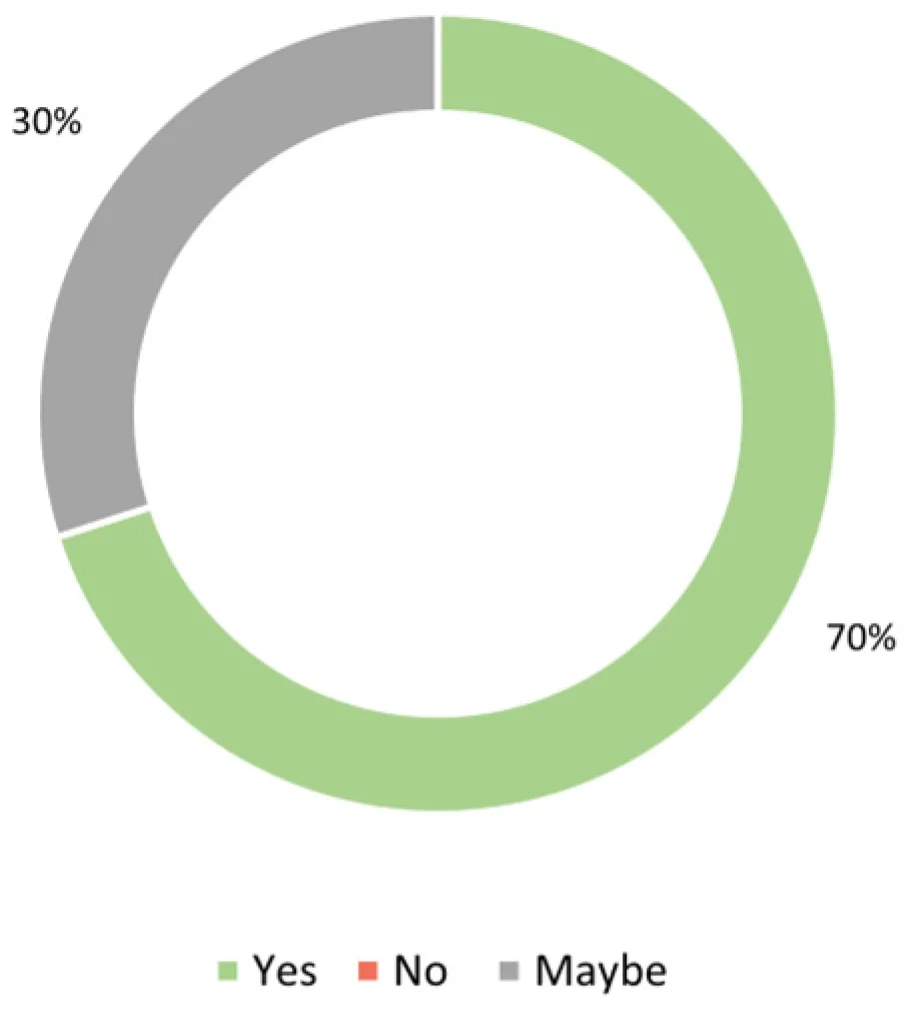
Distribution of responses regarding the intention to continue using the chair in the long term (n = 10).

**Figure 13:**
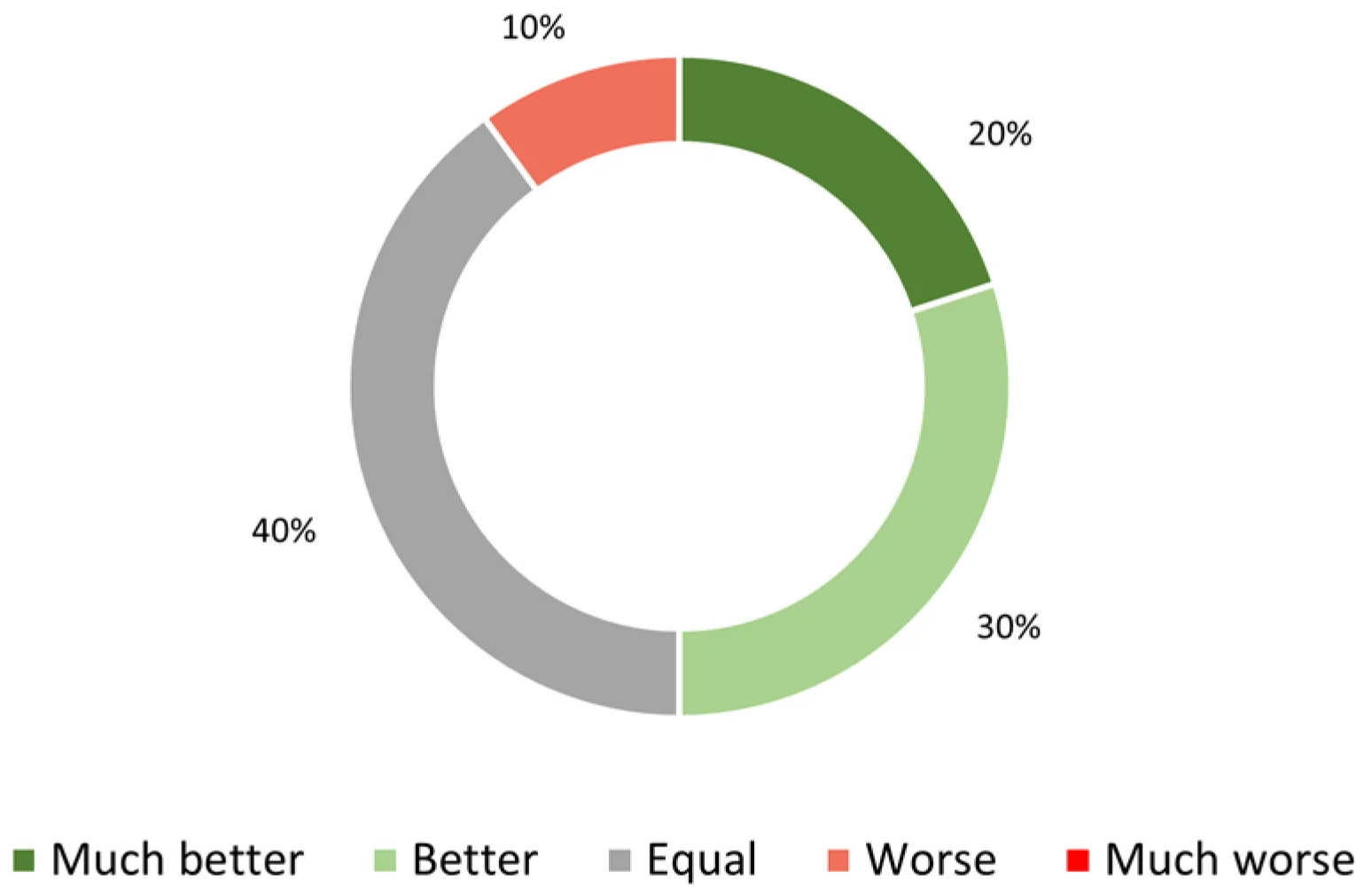
Distribution of participants’ responses comparing their perceived experience with the chair to the one used daily (n = 10).

At the end of the daily use period and after a full week of utilization, all participants completed a technology acceptance questionnaire. Based on the responses provided, a generally positive acceptance of the prototype was observed. All participants (100%) reported using the chair’s feedback to adjust their posture during the validation period (Figure 12 and Figure 13).

**Figure 14:**
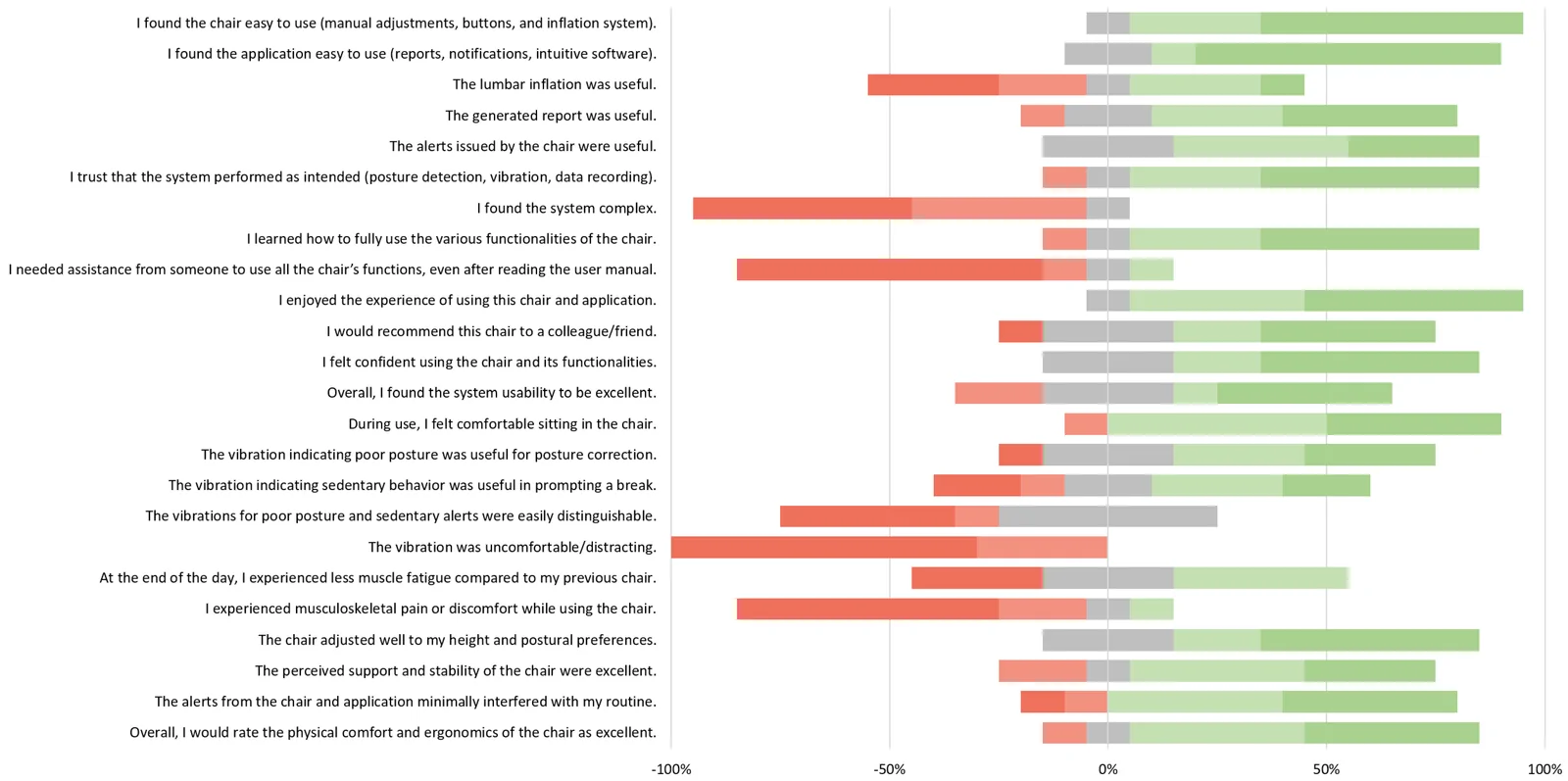
Distribution of participants’ responses to the user acceptance questionnaire, assessed using a Likert scale (1–5), where green represents agreement/strong agreement, grey a neutral position, and red disagreement/strong disagreement (n = 10).

Additionally, 70% of participants indicated that they would continue using the chair in the long term, while 30% expressed uncertainty. Regarding perceived comfort, most participants reported improvements when compared to their usual chair, with 20% indicating a much better experience, 30% better, and 40% reporting no significant change, while only 10% reported a worse experience. Participants highlighted the usefulness of postural feedback for posture correction, as well as the ease of use of both the system and its associated application (Figure 14). Overall comfort was also positively evaluated; however, certain aspects related to the feedback mechanisms, particularly the distinction between different types of vibration alerts, were identified as areas requiring improvement.

Overall, the results demonstrate the feasibility of the proposed concept, indicating a high level of user acceptance while simultaneously identifying concrete opportunities for optimization, particularly in the classification algorithm, feedback system design, and environmental monitoring functionality.

## 4. Discussion

Over recent years, particularly following the COVID-19 pandemic, there has been a significant increase in the prevalence of sedentary behaviour. The expansion of remote work (home office) has contributed substantially to this trend, as ergonomic conditions, both in domestic environments and in offices, are often suboptimal. This situation promotes the adoption of incorrect postures among workers [28]. Additionally, the demands and pressures associated with professional activities have contributed to a reduction in time allocated to physical activity, thereby increasing sedentary behaviour [29].

Several studies support this trend. For instance, Park et al. (2026) [30], using accelerometry analysis, reported that approximately 80 information technology workers spend, on average, 10.76 h per day in sedentary behaviour, of which 3.72 h correspond to prolonged sedentary periods. In contrast, only 221 min of daily physical activity were recorded, with just 21 min classified as moderate-to-vigorous physical activity [30].

Despite the growing body of evidence, there are still no universally established policies regarding maximum sedentary time in the workplace or recommended postures. Nevertheless, the European Union acknowledges the relevance of this issue, particularly in the context of WMSDs, incorporating it into its occupational safety and health strategy [31]. In parallel, policy recommendations have been developed to promote physical activity, including within occupational settings [32].

Prolonged sitting is recognized as a significant risk factor for the development of various conditions, including lower back pain, neck and shoulder pain, spinal misalignment, and other musculoskeletal disorders, as well as metabolic diseases such as diabetes and obesity. According to EU-OSHA, workers engaged in predominantly sedentary tasks should limit sitting time to less than 50% of their working hours. Additionally, regular breaks are recommended, with interruptions of sitting every 20–30 min, including at least 10 min of activity every two hours. In the present study, these guidelines were considered when defining maximum sedentary thresholds [33].

In recent years, several systems have been proposed to monitor and correct workers’ posture using pressure and force sensors. P. Divya Jenifar et al. (2024) [34] developed an approach for seated posture detection aimed at improving comfort and reducing the incidence of lower back pain during prolonged sitting [13].

Based on this context, the present study proposes the development of an integrated and multifunctional system capable of monitoring working posture, alerting for excessive sedentary periods, and assessing environmental parameters in the workplace. The inclusion of environmental monitoring is particularly relevant, given the strong relationship between workplace conditions and posture. Extreme or polluted environments can increase both physical and mental workload, contributing to poorer posture, increased fatigue, drowsiness, musculoskeletal pain, injuries, and headaches [35]. Indeed, evidence suggests that an optimal work environment, characterized by moderate temperature, adequate lighting, effective organization, and ergonomic practices, significantly reduces health risks such as fatigue and musculoskeletal pain, while enhancing productivity and focus [36]. A limitation of this study is that differences between internal and external measurements may arise from factors such as sensor positioning, the microenvironment within the chair, and local airflow variations. Additionally, particle measurements are inherently sensitive to sensor placement, local ventilation, and resuspension phenomena; therefore, these results should be interpreted with caution and not considered a full metrological validation. This is clear given the relative error of almost 40% in PM 2.5 and of 31% in PM 10. For the remainder of the examined variables, the relative error is below 10%, especially for the temperature, where the error is only 1.28%. Therefore, the environmental monitoring module should be interpreted as a preliminary component of the multimodal hardware prototype rather than as a fully validated air quality monitoring system. In particular, the high relative errors observed for PM_2.5_ and PM_10_ indicate that particulate matter measurements are not yet sufficiently reliable for quantitative air quality assessment. These parameters are highly sensitive to sensor positioning, local airflow conditions, and particle resuspension caused by user movement and sitting dynamics. Consequently, PM_2.5_ and PM_10_ results should be considered exploratory in the current prototype. Future developments should focus on optimizing sensor placement, improving airflow exposure to the particulate matter sensor, minimizing interference from the chair structure and fabric, and implementing dedicated calibration procedures before using these variables for practical environmental monitoring.

Environmental parameters were included as a complementary component of the system to support a broader and more holistic assessment of workplace ergonomics. Although posture classification was the primary focus of the study, environmental conditions such as temperature, relative humidity, CO_2_, particulate matter, VOCs, and NOx may influence user comfort, fatigue, alertness, and overall well-being, and are therefore relevant in the context of workplace risk prevention. In the current prototype, however, these parameters were not included as input features for posture classification because their primary purpose was to characterize the workplace environment and validate the environmental monitoring functionality of the chair. Moreover, these parameters were collected at fixed time points during the day, whereas posture classification relied on pressure sensor data acquired during the execution of each posture. Therefore, environmental measurements were not temporally synchronized with each posture sample at the same acquisition rate and were not considered suitable as direct classification features in the current model. However, temperature and relative humidity may influence pressure sensor stability and signal drift. Future work will therefore investigate the use of environmental data, particularly temperature and humidity, as compensation variables for pressure sensor calibration and signal normalization, with the aim of improving robustness under real-world conditions.

Furthermore, proper workstation organization and the use of equipment designed to reduce physical workload play a key role in improving ergonomic conditions. The results obtained from a technology acceptance questionnaire indicate a positive perception among users, who reported ease of use and practical usefulness of the system in their daily work. These findings are consistent with the study by Kim et al. (2024) [37], which highlights that user trust and ease of use are key determinants of technology acceptance.

Finally, it is important to emphasize that the developed system adopts a holistic ergonomic assessment approach, enabling the integrated characterization of both worker posture and the work environment. It represents a non-invasive solution capable of providing real-time feedback, supporting ergonomic assessment, and promoting postural awareness, thereby assisting in improvements in working conditions and potentially contributing to reduced physical strain and enhanced worker well-being [38]. The results indicate that the prototype still requires further refinement before practical deployment. Regarding the pressure sensors, it was observed that their readings may be affected by postures outside the ten predefined classes, as well as by subtle differences between postures with similar pressure-distribution patterns, which may lead to incorrect classification. This is particularly relevant in real-world conditions, where greater inter-user variability, differences in posture execution, and natural interaction with the chair reduce the consistency of the pressure patterns. Although the system achieved an accuracy above 80% under controlled testing conditions, performance decreased to 65.98% during the in-field validation protocol. This reduction highlights the challenge of transferring posture classification models from controlled settings to workplace conditions and indicates that the current prototype should be considered a feasibility prototype rather than a deployment-ready solution. To further contextualize these findings, the naturalistic data collected during the 5-day workplace use period were also analyzed. Unlike the supervised posture-recognition protocol, these data reflect how participants actually used the chair during their normal work activities. The results showed substantial inter-participant variability in posture distribution, confirming that sitting behaviour in office work is highly individual and influenced by task demands, comfort preferences, habitual postural patterns, and the way each user interacts with the chair. Consequently, future iterations of the system should consider adaptive alert strategies, personalized thresholds and feedback modalities that are less prone to habituation [39].

To better understand the landscape of available smart chair solutions and evaluate the performance of the prototype developed in this study, a comparison with the current state of the art is essential. Table 9 presents several smart chair implementations reported in the literature, demonstrating that similar systems have already been developed and investigated by previous researchers. When comparing our results with those reported in earlier studies, the proposed system stands out for its ability to assess a broader range of sitting postures, showing similarities to the approaches described by Jeoung et al. (2021) [40] and Ishac et al. (2018) [39]. Nevertheless, both studies achieved higher classification accuracies than those obtained in the present work. These findings highlight the need for further optimization of the developed system. Potential improvements may include increasing the number of sensors, modifying their placement on the chair, or exploring alternative approaches for the posture classification algorithm. Such modifications could enhance the system’s ability to accurately distinguish between different postural patterns.

Another relevant aspect identified through comparison with previous studies is the number of participants involved in the data collection and model validation processes. Studies reporting higher classification accuracy generally relied on larger and more diverse samples, which contributed to improved model generalizability and performance. Therefore, the relatively small sample size used in this study should be considered a limitation, as it may have affected the robustness of the algorithm and the overall accuracy achieved. Future work should include a larger participant pool to strengthen the reliability and applicability of the proposed system.

Nevertheless, the results demonstrate that the system can identify several clearly distinguishable postures with promising performance, providing a relevant basis for further optimization. Future work should focus on increasing robustness through additional training data, improved feature extraction, additional model training with different algorithms (random forest, support vector machines, k-nearest neighbours, convolutional neural networks) and increased complexity, adaptive or user-specific calibration, inclusion of a greater diversity of spontaneous postures, and continuous validation during unsupervised workplace use.

A limitation of the current approach is the use of a single interpretable decision tree classifier. Although this model is suitable for early-stage embedded implementation, it may not fully capture complex spatial relationships within the pressure matrix, particularly for postures with similar pressure-distribution patterns. Future work should evaluate more robust spatial and ensemble-based models, such as random forests, support vector machines, k-nearest neighbours, and convolutional neural networks, using larger datasets and subject-independent validation strategies.

Furthermore, lumbar inflation did not improve overall classification performance in controlled conditions, with accuracy decreasing from 82.13% without inflation to 79.87% with inflation. This suggests that, in the current prototype, the inflation mechanism may introduce additional variability in the backrest sensor readings by modifying the contact area and pressure distribution between the user and the chair. More specifically, inflation may deform the fabric where the backrest sensors are embedded, particularly around sensors S3 and S4, generating artificial pressure variations or false contact patterns. Therefore, this effect should be considered a mechanical and sensing integration limitation of the current prototype, rather than an isolated anomaly. Future versions of the system should implement dynamic compensation strategies for inflation-induced pressure changes, such as baseline correction at each inflation level, pressure normalization before classification, inflation-state-dependent calibration, and/or inclusion of the inflation level as an additional input variable to the classification model. Future work should include a dedicated pressure-sensor characterization protocol, including controlled calibration, assessment of temperature drift, creep behaviour, repeatability, and long-term stability under representative thermal and mechanical loading conditions. These procedures will be essential before translating the prototype into a deployment-ready system. A further limitation is that the current model operates as a closed-set classifier. Thus, postures outside the ten predefined classes are assigned to the closest known class, which may lead to misclassification during naturalistic use. Future versions should include an “unknown/other” posture class or confidence-based rejection strategy.

## 5. Conclusions

This study introduces a novel and integrative approach for ergonomic risk assessment in seated positions, combining pressure sensing technology with automated posture identification. The proposed system demonstrates significant potential as an advanced tool for objective, continuous, and non-invasive monitoring of workplace ergonomics, addressing existing gaps in traditional assessment methods. At this stage, the system should be regarded as a feasibility prototype rather than a deployment-ready solution. Future work will focus on improving classification robustness under real-world conditions before practical implementation.

By enabling real-time feedback and comprehensive characterization of user posture, this approach represents a meaningful step toward more proactive and data-driven ergonomic interventions. Moreover, this work lays the foundation for the development of intelligent ergonomic systems that not only assess risk but also actively contribute to behavioural change.

Nevertheless, further work is required to improve system accuracy in both ergonomic and environmental parameter measurements. Future developments should focus on enhancing robustness and reliability under real-world conditions. It is also expected that this approach may be considered by occupational health and safety teams as a valuable tool to support ergonomic assessment, promote postural awareness, and contribute to the mitigation of musculoskeletal risk factors in office environments, ultimately helping to reduce absenteeism associated with these conditions.

## Figures and Tables

**Table 1:** Extracted features and explanations.

Feature Name	Explanation
CoP_X	COP in Axis X in cm
CoP_Y	COP in Axis Y in cm
Q1_Pmax	Maximum Pressure in Q1 kg/cm2
Q2_Pmax	Maximum Pressure in Q2 kg/cm2
Q3_Pmax	Maximum Pressure in Q3 kg/cm2
Q4_Pmax	Maximum Pressure in Q4 kg/cm2
Q1_PM	Average Pressure in Q1 kg/cm2
Q2_PM	Average Pressure in Q2 kg/cm2
Q3_PM	Average Pressure in Q3 kg/cm2
Q4_PM	Average Pressure in Q4 kg/cm2
Q1_%P	Relative Pressure in Q1 in relation to all 4 quadrants %
Q2_%P	Relative Pressure in Q2 in relation to all 4 quadrants %
Q3_%P	Relative Pressure in Q3 in relation to all 4 quadrants %
Q4_%P	Relative Pressure in Q4 in relation to all 4 quadrants %
Q1_A	Contact Area in Q1 in cm2
Q2_A	Contact Area in Q2 in cm2
Q3_A	Contact Area in Q3 in cm2
Q4_A	Contact Area in Q4 in cm2
S1	Sensor embedded in Backrest at Position S1
S2	Sensor embedded in Backrest at Position S2
S3	Sensor embedded in Backrest at Position S3
S4	Sensor embedded in Backrest at Position S4
S5	Sensor embedded in Backrest at Position S5
S6	Sensor embedded in Backrest at Position S6
Q1_A8×8	Area within Q1 in an 8 cm by 8 cm Square
Q2_A8×8	Area within Q2 in an 8 cm by 8 cm Square

**Table 2:** Exposure limit values for the environmental parameters considered, serving as reference thresholds for air quality and occupational exposure assessment.

Parameter	Reference	Time-Weighted Average Exposure Limit Value
Temperature (T/°C)	ISO 7730:2025 [16]	20–24
Relative Humidity (%)	ISO 7730:2025 [16]	40–60
Carbon Dioxide (ppm)	NP 1796:2014 [17]	5000
Nitrogen Oxides (NOx) (ppm)	NIOSH [18]	25
Volatile Organic Compounds (COV) (ppm)	OSHA [19]	600
Respirable Particles (PM_2.5_) (mg/m^3^)	NP 1796:2014 [17]	3
Inhalable Particles (PM_10_) (mg/m^3^)	NP 1796:2014 [17]	10

**Table 3:** Ergonomic risk level associated with each posture, categorized into three classes: acceptable, moderate, and critical.

Risk Level	P1	P2	P3	P4	P5	P6	P7	P8	P9	P10
Acceptable	X									
Moderate			X		X	X				
Critical		X		X			X	X	X	X

**Table 4:** Composition of the training and validation datasets, showing the number of participants and data entries included in a representative cross-validation fold.

Subset	Number of Participants	Number of Entries
Training	66	1311
Validation	17	329

**Table 5:** Distribution of samples among the different posture classes.

Position	P1	P2	P3	P4	P5	P6	P7	P8	P9	P10	Empty
Number of entries	124	125	124	126	125	125	124	125	127	127	388

**Table 6:** Hyperparameter settings employed during model development and evaluation.

Hyperparameter	Value	
	Aggressive	Conservative
Criterion	Gini and Shannon Entropy	Gini
Maximum Depth	8	10 or 15
Minimum Sample Split	2 or 5	10
Minimum Samples Leaf	1 or 5	5 or 10
Maximum Leaf Nodes	120	60 or 100
Minimum Impurity Decrease	0.0	0 or 0.001

**Table 7:** Results of the position classification algorithm with and without inflation, demonstrating higher overall accuracy in the non-inflated model (82.13% vs. 79.87%) (▼ and ▲ symbols indicate that the value is lower or higher, respectively, compared to the same value in a different condition).

	Without Inflation									
	P1	P2	P3	P4	P5	P6	P7	P8	P9	P10
Precision	▲73.21%	▼85.71%	▲78.33%	▲75.68%	▼67.74%	▲91.67%	▼84.21%	▼77.78%	▲89.23%	▲98.36%
Recall	▼66.13%	▲87.10%	▲75.81%	▲88.89%	▼67.74%	▲88.71%	▼77.42%	▼79.03%	▲92.06%	▲95.24%
F1-Score	▼69.49%	▼86.40%	▲77.05%	▲81.75%	▼67.74%	▲90.16%	▼80.67%	▼78.40%	▲90.63%	▲96.77%
	With Inflation									
	P1	P2	P3	P4	P5	P6	P7	P8	P9	P10
Precision	▼72.13%	▲96.30%	▼70.31%	▼66.67%	▲68.06%	▼90.16%	▲88.33%	▲82.26%	▼75.36%	▼95.16%
Recall	▲70.97%	▼82.54%	▼72.58%	▼69.84%	▲77.78%	▼85.94%	▲84.13%	▲80.95%	▼81.25%	▼92.19%
F1-Score	▲71.54%	▲88.89%	▼71.43%	▼68.22%	▲72.59%	▼88.00%	▲86.18%	▲81.60%	▼78.20%	▼93.65%

**Table 8:** Results of the Er between the internal PCB and the external PCB.

	T/°C	HR (%)	COV	PM_2.5_	PM_10_	NO_X_	CO_2_
Er (%)	1.28	7.97	14.91	39.80	31.22	7.45	9.34

**Table 9:** Summary of previous smart chair approaches and comparison with the proposed prototype.

Reference	Nº of Subjects	Sensor Type	Sensor Location	Algorithm	Nº of Postures	Accuracy
Wan et al. (2021) [41]	10	Pressure Sensor Array	On top of seat	SVM	4	89.6%
Jeoung et al. (2021) [40]	36	FSR and Infrared Reflective Distance Sensors	6 FSR on seat cushion and 6 distance sensors on backrest	k-Nearest Neighbours	11	92.0%
Ishac et al. (2018) [39]	20	Conductive Fabric Pressure sensing Array	Cushion placed on top of backrest	Empirical Threshold	11	98.1%
Kim et al. (2018) [42]	10	Pressure sensor mat	Inside chair seat cushion	CNN	5	95.3%
Roh et al. (2018) [43]	9	Load Cell	4 on seat plate	SVM	6	97.2%
This Study	10	Pressure Sensors	Embedded in the Backrest and Mat in the Seat	Decision Tree	10	65.98%

## Data Availability

Data are unavailable due to privacy or ethical restrictions.

## Abbreviations

The following abbreviations are used in this manuscript:

AI	Artificial Intelligence
CO_2_	Carbon Dioxide
COP	Centre of Pressure
CV	Cross-validation
EU-OSHA	European Agency for Safety and Health at Work
FSRs	Force-sensitive Resistors
PM 10	Inhalable Particles
ML	Machine Learning
NOx	Nitrogen Oxides
Pa	Pressure Values
PM 2.5	Respirable Particles
VOCs	Volatile Organic Compounds
WMSDs	Work-related Musculoskeletal Disorders
WHO	World Health Organization
